# Forests buffer the climate‐induced decline of body mass in a mountain herbivore

**DOI:** 10.1111/gcb.15711

**Published:** 2021-06-03

**Authors:** Rudolf Reiner, Andreas Zedrosser, Hubert Zeiler, Klaus Hackländer, Luca Corlatti

**Affiliations:** ^1^ Institute of Wildlife Biology and Game Management University of Natural Resources and Life Sciences Vienna Austria; ^2^ Berchtesgaden National Park Berchtesgaden Germany; ^3^ Department of Natural Sciences and Environmental Health University of South‐Eastern Norway Bø i Telemark Norway; ^4^ Dellach Austria; ^5^ Chair of Wildlife Ecology and Management University of Freiburg Freiburg Germany

**Keywords:** body mass, chamois, climate change, forest, *Rupicapra rupicapra*, temperature, ungulate

## Abstract

Climate change is known to affect key life‐history traits, such as body mass, reproduction, and survival in many species. Animal populations inhabiting mountain habitats are adapted to extreme seasonal environmental conditions but are also expected to be especially vulnerable to climate change. Studies on mountain ungulates typically focus on populations or sections of populations living above the tree line, whereas populations inhabiting forested habitats are largely understudied. Here, we investigate whether forested areas can mitigate the impact of climatic change on life‐history traits by evaluating the interactive effects of temperature and habitat characteristics on body mass variation in the Alpine chamois *Rupicapra rupicapra rupicapra*. We examined data of 20,573 yearling chamois collected from 1993 to 2019 in 28 mountain ranges in the Austrian Eastern Alps, characterized by different proportion of forest cover. Our results show that the temporal decline of chamois body mass is less pronounced in areas with greater proportion of forest cover. For chamois living in forest habitats only, no significant temporal change in body mass was detected. Variation in body mass was affected by the interaction between density and snow cover, as well as by the interaction between spring temperatures and forest cover, supporting the role of forests as thermal buffer against the effects of increasing temperatures on life‐history traits in a mountain ungulate. In turn, this study suggests a buffering effect of forests against climate change impacts. Assessments of the consequences of climate change on the life‐history traits and population dynamics of mountain‐dwelling species should thus consider the plasticity of the species with respect to the use and availability of different habitat types.

## Graphical abstract

Climate change is known to affect key life history traits, such as reproduction, survival and body mass in many species. Our results show that the temporal decline in body mass of Alpine chamois *Rupicapra rupicapra rupicapra* is less pronounced in areas with greater proportion of forest cover, therefore suggesting a buffering effect of forests against climate change impacts. Assessments of the consequences of climate change on the life history traits and population dynamics should thus consider the plasticity of the species with respect to the use and availability of different habitat types (Drawings: Hubert Zeiler).

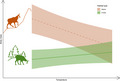

## 1 INTRODUCTION

Awareness of the impacts of global change on organisms and ecosystems has increased in recent decades (Walther et al., 2002). For example, changing climatic conditions affect organisms’ physiology, morphology (Musolin, 2007; Ozgul et al., 2009), and behavior (Mason, Stephens, et al., 2014; Van Buskirk et al., 2012), which, in turn, affect life‐history traits, such as reproductive success, survival, and ultimately population dynamics (Betts et al., 2018; Ozgul et al., 2010). Several studies have investigated the effects of climatic parameters on demographics and key traits of a variety of taxa in different ecosystems, from the negative effect of increasing temperatures on bird distribution (Erasmus et al., 2002), breeding phenology (Wang et al., 2002), the decrease in marine animal biomass (Bryndum‐Buchholz et al., 2019), to elevational and latitudinal range shifts in many plant and animal species (Parmesan & Yohe, 2003). A common general conclusion is that the effect of climate change may cause massive reduction, or even extinction, of entire orders of plants, insects, and mammals (Pio et al., 2014).

Increasing ambient temperatures, in particular, may severely affect key life‐history traits such as body mass, an important indicator of individual fitness in animals (e.g., Gaillard et al., 2000; Stewart et al., 2005). A global trend of decreasing body mass in the context of global warming has been reported for a growing number of taxa (Gardner et al., 2011), including birds (Salewski et al., 2010; Yom‐Tov, 2001), mammals (Ozgul et al., 2010; Rode et al., 2010), and ectotherms (Daufresne et al., 2009; Genner et al., 2010), in both terrestrial and aquatic ecosystems. Changes in temperature may induce a decline in body mass either directly or indirectly (Gardner et al., 2011). For example, rising temperatures due to climate change may increase costs of thermoregulation which, in turn, may lead to a decline in body mass, as shown in Southern pied babblers *Turdoides bicolor* (du Plessis et al., 2012) and lizards *Varanus* spp. (Kearney et al., 2009). Likewise, higher temperatures may reduce resource availability, leading to a decline in reproduction and survival rates (Clutton‐Brock & Pemberton, 2004; Lenarz et al., 2009; Pettorelli et al., 2007; Ruprecht et al., 2016), thus affecting population dynamics (e.g., in Canadian lynx *Lynx canadensis*: Yom‐Tov et al., 2007).

Temperature increase due to climate change is particularly marked at high elevations and latitudes (Diaz & Bradley, 1997; Turco et al., 2015). Wildlife populations inhabiting these environments are adapted to cope with harsh and seasonally variable conditions (Aublet et al., 2009; McCann et al., 2013); thus, they are expected to be especially vulnerable to rising temperature in the context of climate change (Jenouvrier et al., 2009; Parmesan, 2006). For example, body mass of moose *Alces alces* living at high latitudes has decreased in relation to long‐term temperature increases in spring and early summer (Herfindal et al., 2006). In addition, changes in vegetation green‐up may create mismatches between resource availability and birth date, thereby decreasing foraging opportunities during a critical time of the year and impacting population demography, as observed in several large herbivores at high altitudes (e.g., Lovari et al., 2020; Plard et al., 2014; Rattenbury et al., 2018).

The Alpine chamois *Rupicapra rupicapra rupicapra* is the most abundant mountain ungulate of the European Alps (Corlatti et al., 2011), and is morphologically and physiologically adapted to cold climates (Ascenzi et al., 1993). When faced with temporary increases in temperature, individuals show behavioral thermoregultory adaptions, for example, by moving to higher elevations (Mason, Stephens, et al., 2014) or by shifting activity rhythms to more favourable times of day (Grignolio et al., 2018). In contrast, long‐term increases in ambient temperatures have resulted in decreases in chamois body mass (Mason, Apollonio, et al., 2014; Rughetti & Festa‐Bianchet, 2012). Most studies on chamois have focused on populations living in mountainous areas above the tree line; however, evidence suggests that montane and subalpine forests are also part of the species’ native habitat and that the current, predominantly alpine distribution may largely be a consequence of post‐Neolithic hunting pressure (Baumann et al., 2005). Therefore, chamois are fairly plastic in their habitat choice, and in the presence of steep, rocky terrain (von Elsner‐Schack, 1985), populations can permanently inhabit forested areas even at low elevations. In turn, the ecological plasticity of chamois suggests that long‐term effects of climate change on body mass may differ in relation to the environmental settings, as life‐history responses to climatic conditions may vary among habitats (Chirichella et al., 2021; Loison et al., 1999). Forests, for example, may buffer phenotypical traits against adverse environmental conditions (Betts et al., 2018; Melin et al., 2014), as climatic conditions underneath the forest canopy are typically less extreme compared to the open landscape (Ewers & Banks‐Leite, 2013; Mora et al., 2011).

To our knowledge, no study has yet investigated the interactive effects of temperature and habitat characteristics on body mass variation in ungulates. Here, we investigate the effect of forest cover on variations in the body mass of chamois in the Eastern Alps of Austria. We take advantage of a large dataset (*n* = 20,573) of individuals harvested over a 27‐year period (1993–2019) in habitats with different proportions of forest cover, to test hypotheses on the habitat‐specific effects of climatic conditions on the body mass of yearling (1‐year old) chamois, that is, the age class most affected by environmental conditions (Mason, Apollonio, et al., 2014; Rughetti & Festa‐Bianchet, 2012; Willisch et al., 2013). Specifically, we hypothesize that the body mass of yearling chamois (i) changes over time (cf. Mason, Apollonio, et al., 2014; Rughetti & Festa‐Bianchet, 2012) in relation to forest cover and that (ii) the effect of spring and summer temperatures on body mass varies with the amount of forest cover. Accordingly, after accounting for potential confounding factors such as population density and snow depth, we predict that body mass of yearling chamois is significantly related to (a) the interaction between year and forest cover, that is, there is a less pronounced decrease in body mass with increasing forest cover and to (b) the interaction between spring–summer temperatures and forest cover, that is, there is a less pronounced effect of increasing spring–summer temperatures on body mass with increasing forest cover.

## 2 MATERIALS AND METHODS

### 2.1 Study area and populations

**FIGURE 1:**
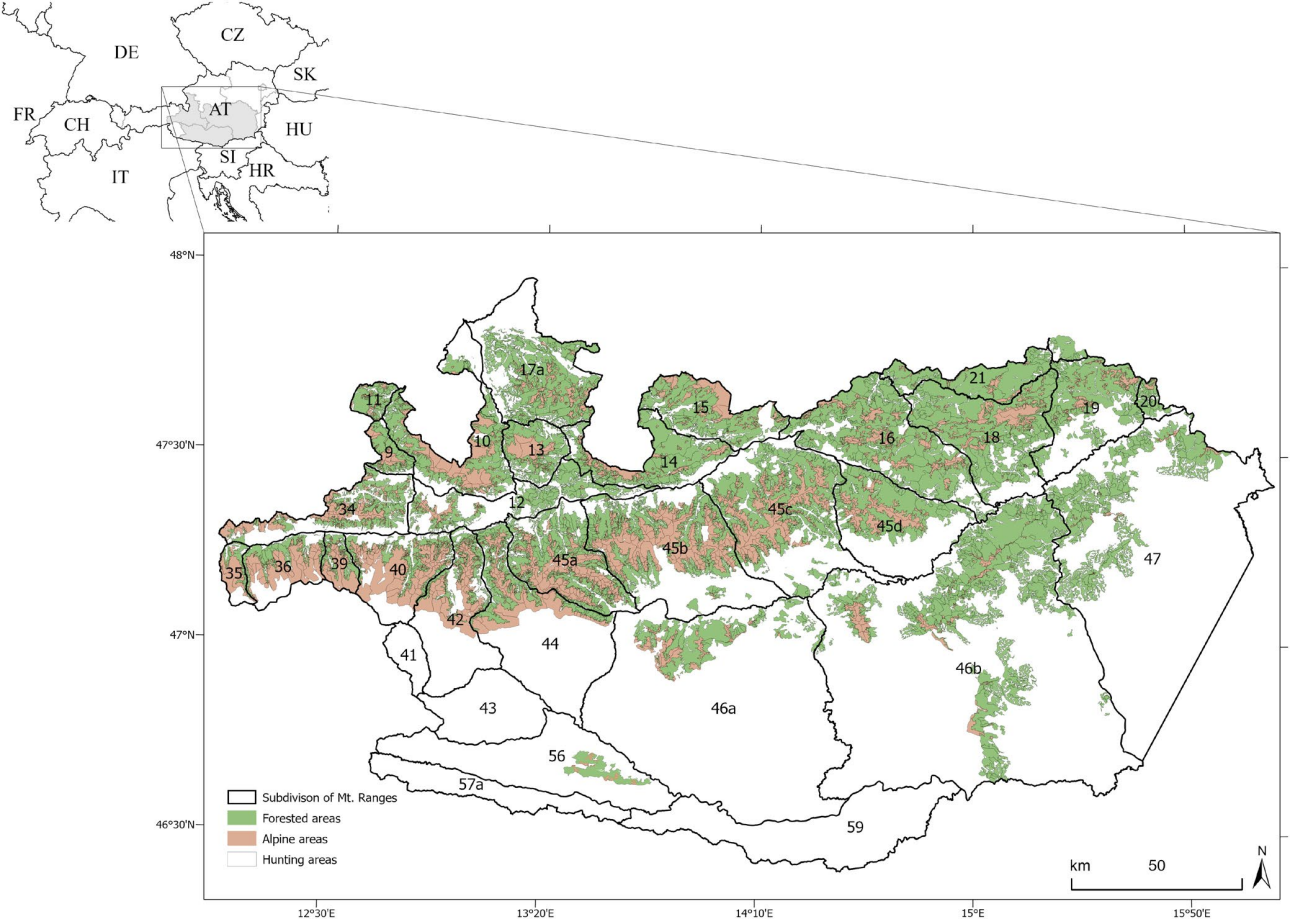
The study area in the provinces of Carinthia, Salzburg, and Styria, Austria. Numbers and black lines correspond to the geographic subdivision of the Eastern Alps. Mountain range IDs between 9 and 21 belong to the Northern Limestone Zone, 35–47 to the Central Alps, ≥56 to the Southern Limestone Zone. Colored areas show suitable habitat for chamois within hunting areas, which are defined as the sum of all open Alpine areas (i.e., Alpine meadows, sparsely vegetated areas, and bare rocks) and all forested areas (i.e., broad leaved, coniferous, and mixed forests) for hunting areas with chamois harvest during the study period

The study area was located in the Austrian part of the Eastern Alps (approximately 46°38′N–47°50′N, 12°04′E–16°00′E) and extends over 13,600 km^2^ with an altitudinal range from 300 to 3500 m a.s.l. (Figure 1). The study area includes all hunting areas where chamois have been shot in the provinces of Salzburg and Styria, as well as some hunting areas in the province of Carinthia. Within the study area, hunting areas were grouped according to mountain ranges (Mason, Apollonio, et al., 2014; Mason et al., 2011), following the geographic subdivision of the Eastern Alps (Grassler, 1984; Table S1). The grouping according to mountain ranges coincides with chamois (sub)populations (*n* = 28) with only limited dispersal among (sub)populations (cf. Reiner, 2015; Reiner et al., 2020).

The predominant substrate type is calcareous in the northern (*n* = 12 mountain ranges) and southern limestone zones (*n* = 1), and siliceous in the central Alps (*n* = 15; Grassler, 1984; Figure 1). The predominant tree species in all mountain ranges is Norway spruce *Picea abies*. In mountain ranges with calcareous substrate type, other dominant tree species are beech *Fagus sylvatica*, Scots pine *Pinus sylvestris*, and silver fir *Abies alba* at lower elevations, and dwarf mountain pine *Pinus mugo* at higher elevations. On siliceous substrate, Norway spruce, silver fir, and European larch *Larix decidua* are the dominant tree species. At higher elevations, green alder *Alnus viridis* and dwarf mountain pine are common. Above the tree line (approx. >1800 m a.s.l.) the habitat consists mainly of alpine meadows, sparsely vegetated areas, and bare rocks at the highest elevations throughout the study area. Diet of Alpine chamois mainly consists of graminoids (Poaceae and Cyperaceae), herbs, sedges, and also woody plants (Anderwald et al., 2016). Negligible kid mortality might be caused by the golden eagle (*Aquila chrysaetos*; Bertolino, 2003). No stable permanent populations of large carnivores are currently present in the study area.

### 2.2 Body mass

To investigate the potential drivers of temporal variation in chamois body mass, we used long‐term harvest data of yearlings collected routinely by local hunting authorities between 1993 and 2019 (15 mountain ranges) and 1998 and 2019 (13 mountain ranges), respectively (Table S2). Within each hunting area, quotas are set for specific sex and age classes, and the duration of the hunting season is from July 16 to December 15 in Salzburg, and from August 1 to December 31 in Styria and Carinthia. Yearlings are hunted solely on the basis of numerical quotas, that is, not in relation to physical or sex characteristics, and biometric measurements were collected for all hunted individuals according to provincial hunting regulations (Reiner et al., 2020). For each harvested individual, body mass (eviscerated, without head, with skin) was recorded with a precision of 0.5 kg. Chamois were classified as yearlings based on horn size and dentition (Schröder & von Elsner‐Schak, 1985). Since we were interested in the effect of spring and summer temperatures on variations in body mass, we only included data of chamois harvested after August 31 in our analysis. Overall, we collected body mass data on 20,573 individuals (11,018 female and 9555 male yearlings).

### 2.3 Explanatory variables

**FIGURE 2:**
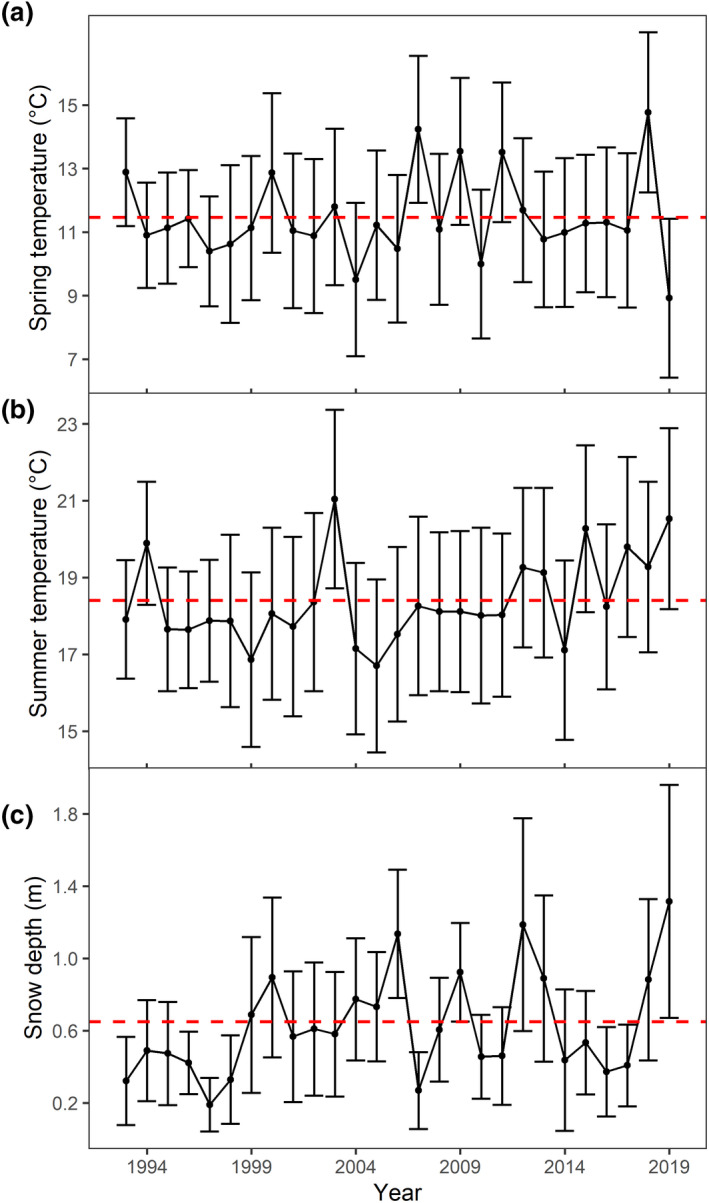
Mean spring (April–May) temperature (a), mean summer (June–August) temperature (b), and mean snow depth (c) in mountain ranges in the study area, between 1993 and 2019. The red horizontal lines indicate the average value of the corresponding climate variable. Bars indicate standard deviations across mountain ranges

To investigate variation in yearling body mass, our explanatory variables included sex, year of harvest, hunting date, mountain range, climatic variables, population density, and forest cover of mountain range. The sex of each harvested individual, in addition to year, day of hunting (Julian day), and the hunting area are routinely collected by local hunting authorities alongside body mass data. Climatic data included mean daily maximum temperature (in °C) in spring (April–May) and in summer (June–August) in the current year [*t*] and in the year of birth [*t* − 1] (cf. Rughetti & Festa‐Bianchet, 2012), and yearly average daily snow depth (in cm) in early winter (October–December) of the preceding year [*t* − 1], in winter (January–February) and late winter/spring (March–May) of the current year [*t*], as these variables are expected to exert an important effect on chamois yearling body mass (Loison et al., 1999; Mason, Apollonio, et al., 2014; Rughetti & Festa‐Bianchet, 2012; Willisch et al., 2013). Climatic data were obtained from the Central Institution for Meteorology and Geodynamics (ZAMG), Austria, and had a spatial resolution of 1 × 1 km. Daily temperatures during our study period were extracted from the SPARTACUS dataset (Hiebl & Frei, 2016), which uses data interpolation from multiple meteorological stations and digital elevation models while accounting for obstruction of air‐flow due to topographic features (Frei, 2014). With this approach, local weather phenomena, such as cold air pools and warm air layers, which are especially relevant in mountainous areas, can be accounted for more effectively than by using simple interpolations based on Euclidian distance between meteorological stations (Hiebl & Frei, 2016). Daily snow depth data were also provided by ZAMG (see Table 1 for more details on climatic variables). For all climatic variables, means over each mountain range were used in further analyses. The temporal trend of spring and summer temperatures, and winter precipitation is shown in Figure 2.

**TABLE 1:** Definitions and descriptions of environmental variables used to evaluate temporal variation in body mass of yearling Alpine chamois in Salzburg, Styria, and Carinthia, Austria, from 1993 to 2019

Environmental variable	Definition	Description
SnowD_ewi	Mean daily snow depth of October, November, and December at year [*t* − 1] (cm)	Snow depth during early winter at [*t* − 1]
SnowD_wi	Mean daily snow depth of January and February at year [*t*] (cm)	Snow depth during winter at [*t*]
SnowD_lwi	Mean daily snow depth of March, April, and May at time [*t* − 1] (cm)	Snow depth during late winter/spring at [*t*]
TSpr_*t* − 1	Mean daily maximum air temperature of April and May in the year of birth [*t* − 1] (°C)	Spring temperature at [*t* − 1]
TSum_*t* − 1	Mean daily maximum air temperature of June, July and, August at [*t* − 1] (°C)	Summer temperature at [*t* − 1]
TSpr_*t*	Mean daily maximum air temperature of April and May (°C)	Spring temperature at [*t*]
TSum_*t*	Mean daily maximum air temperature of June, July and, August (°C)	Summer temperature at [*t*]
Density	Number of harvested chamois at time [*t*]	Proxy of chamois density at [*t*]
Density_*t* − 1	Number of harvested chamois at time [*t* − 1]	Proxy of chamois density at [*t* − 1]
%forest	Relative size of hunting areas which is covered by forests within each mountain range	Forest cover

Population density plays an important role in body mass dynamics of ungulates (Gordon et al., 2004; Morellet et al., 2007; Solberg et al., 1999). Because population size data were not available for all populations, we used the yearly number of harvested chamois of all age classes in relation to suitable habitat (in km^2^; see below for definition) within each mountain range as a density index. This density index is positively and significantly correlated with the population abundance estimates available for two populations included in this study (Reiner et al., 2020; Figure S1).

To investigate the potential effect of forest cover on the effect of increasing ambient temperatures on chamois body mass, we estimated the relative area covered by forest within each mountain range (hereafter ‘forest cover’). The overall suitable chamois habitat was defined as the sum of all open Alpine areas (i.e., Alpine meadows, sparsely vegetated areas and bare rocks) and all forested areas (i.e., broad leaved, coniferous, and mixed forests; Krofel et al., 2013; Reiner et al., 2020; Zeiler, 2012) for hunting areas with chamois harvest during the study period (Figure 1). Forest cover was calculated using the Corine Landcover data (Copernicus Land Monitoring Service, 2018) in ArcGIS Pro 2.6 (ESRI Inc., 2020). Mean forest cover of mountain ranges was 70.6%, ranging between 24.1% and 99.0% (Table S1). A comparison between forest cover in 1990 (the available dataset closest to the beginning of the study period) and 2018 did not reveal major changes in land cover (≤4%; Figure S2), and data inspection suggested that these changes were mainly due to increases in the geometric accuracy of the satellite data (≤50 m in 1990, ≤25 m in 2000–2012, and ≤10 m in 2018). We thus assumed consistency in forest cover throughout the study period and used the most recent land cover data with the highest resolution for statistical analyses.

### 2.4 Statistical analysis

To address the first hypothesis, we investigated the data for the presence of a temporal change in chamois body mass as a function of forest cover. We fitted a ‘time‐dependent’ linear mixed model (LMM) with yearling body mass as the response variable with Gaussian conditional distribution, year, sex, and Julian day as explanatory variables, and mountain range as a random intercept (*n* = 28). The random structure allowed us to consider regional differences while accounting for within‐population correlation in body mass values (Davies et al., 2006; Dormann et al., 2007). Sex was fitted as an additive binary term, as preliminary inspection of data did not reveal differences in body mass time‐variation between yearling males and females. Julian day was modeled with a quadratic effect to account for the nonlinear variation of body mass over time (Figure S3). Next, we refitted the same model, including the interaction year × forest cover as an additional explanatory variable. The two models were compared with a likelihood ratio test to inspect for the occurrence of a potential buffering effect of forest cover on temporal variation in body mass.

To address the second hypothesis, we investigated which combination of intrinsic or extrinsic variables best explained the variation in yearling body mass at different proportional amounts of forest cover. We first fitted a ‘global’ LMM with yearling body mass as the response variable with Gaussian conditional distribution, and as explanatory variables the interactions of forest cover with all environmental factors (snow depth during early winter at [*t* − 1], snow depth during winter at [*t*], snow depth during late winter/spring at [*t*], spring temperature at [*t* − 1], spring temperature at [*t*], summer temperature at [*t* − 1], summer temperature at [*t*]; Table 1), and population density at [*t*]. Sex and quadratic Julian day were fitted as additive covariates while mountain range (*n* = 28) and year (*n* = 27) were fitted as random intercepts to account for repeated measurements within population and year. Prior to data analysis, all continuous explanatory variables were *z*‐transformed within mountain ranges, to make regression coefficients comparable (Zuur et al., 2009). In the linear predictors, we did not include variables with a Pearson correlation coefficient ≥0.7 (Dormann et al., 2013) and kept variables with variance inflation factors (VIFs) <3 (Zuur et al., 2009) to avoid multicollinearity.

Starting from the global model, we fitted a set of simpler models by testing body mass against all possible combinations of the predictor variables with the *dredge* function in the package *‘MuMin’* (Barton, 2020), and retained as candidates all the models with ΔAIC (Akaike's information criterion, Akaike, 1973) ≤2 (Burnham & Anderson, 2002). When comparing models with different systematic structures, we used maximum likelihood estimation, and the models retained in the final set were refitted using restricted maximum likelihood estimation (Zuur et al., 2009). The candidate models were averaged using the package *‘MuMin’* (Barton, 2020) to obtain final parameter estimates (Burnham & Anderson, 2002).

For all models (i.e., temporal change in chamois body mass, global model, and final candidate models), we checked for normality and homogeneity of the conditional distributions by inspecting standardized residuals against fitted values (Zuur et al., 2009; Figure S4). All analyses were conducted using the software R 3.6.1 (R Core Team, 2020) in R Studio 1.2.5001 (RStudio Team, 2019). The packages *‘splines’* (R Core Team, 2020) and *‘lme4’* (Bates et al., 2015) were used for fitting LMM’s with nonlinear effects. Marginal effects were visualized using the package *‘ggplot2’* (Wickham, 2016). To check whether the results depend on the chosen geographical subdivision, the same model was fitted using a different subdivision (i.e., *n* = 49 chamois management units—a combination of several adjacent hunting areas for management purposes, which are in most cases smaller than mountain ranges). Additionally, we checked if results changed when only the period between 1998 and 2019 was considered (i.e., the years where data for all mountain ranges were available).

## 3 RESULTS

**FIGURE 3:**
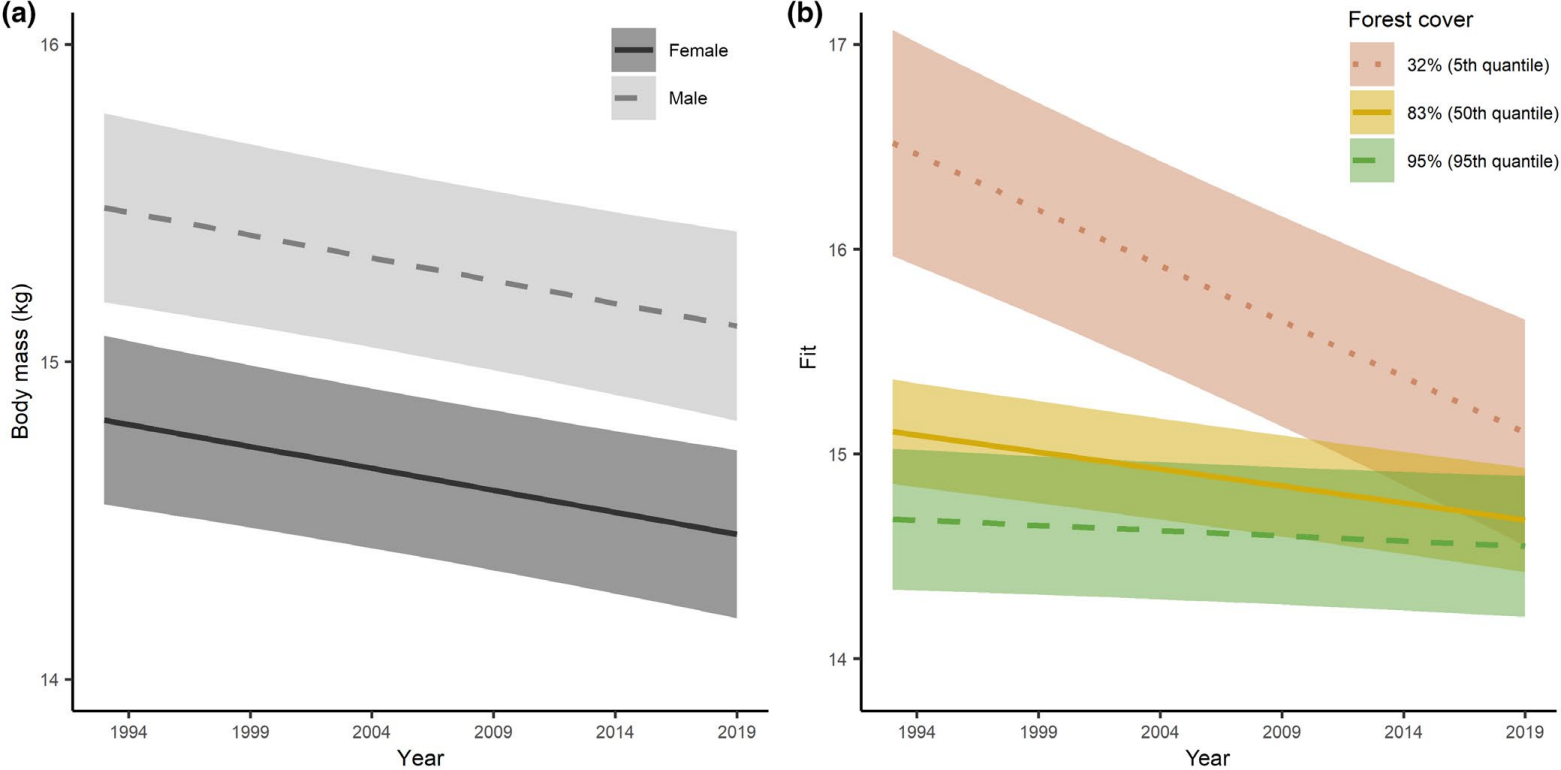
Eviscerated body mass of yearling chamois males (light gray, dashed line) and females (dark gray, solid line) harvested in the provinces of Carinthia, Salzburg, and Styria, Austria, between 1993 and 2019 as a function of year (a) and of the interaction between year and forest cover for both sexes (b) (all other terms of the model are kept at their mean values). The levels of forest cover correspond to the 5th (32% forest cover; red dotted line), 50th (83% forest cover; yellow solid line), and 95th (95% forest cover; green dashed line) quantiles. Shaded areas indicate 95% confidence intervals

Mean body mass was 14.3 ± 2.6 kg and 14.9 ± 2.8 kg for female and male yearlings, respectively, and decreased between 1993 and 2019 (from 14.7 ± 2.7 kg to 13.9 ± 2.6 kg in females, from 15.0 ± 2.7 kg to 14.5 ± 2.7 kg in males). The model fitted to investigate the temporal change in chamois body mass (males: conditional *R* ^2^ = 0.086, marginal *R* ^2^ = 0.013; females: conditional *R* ^2^ = 0.080, marginal *R* ^2^ = 0.010) revealed a significant negative correlation between year and body mass (Figure 3a). When adding the interaction year × forest cover to the model (conditional *R* ^2^ = 0.090, marginal *R* ^2^ = 0.040), the estimates for the interactive effect clearly suggested a difference in the temporal change on body mass as a function of forest cover (Table 2; Figure 3b; likelihood ratio test *p* < 0.001). Spring and summer temperature showed an increasing trend during the study period, with a cluster of high summer temperatures after 2011 (Figure 2a,b).

**TABLE 2:** Mixed model fitted to investigate temporal variation in the body mass of yearling chamois harvested in Salzburg, Styria, and Carinthia, 1993–2019. The table reports variables used in the analysis, beta estimates, standard errors (SE), *t*‐score (*t*), upper and lower 95% confidence interval (CI) and the *p* value

Variable	Estimate	SE	*t*	CI_0.025_	CI_0.975_	*p*
Intercept	13.43	0.15	92.46	13.15	13.72	<0.001
Sex (male)	0.60	0.04	16.36	0.52	0.67	<0.001
Julianday^2^	1.24	0.12	9.98	1.00	1.48	<0.001
Year	−0.12	0.02	−6.53	−0.16	−0.09	<0.001
%forest	−0.32	0.10	−3.33	−0.52	−0.13	<0.001
Year × %forest	0.10	0.02	5.01	0.06	0.14	<0.001

**FIGURE 4:**
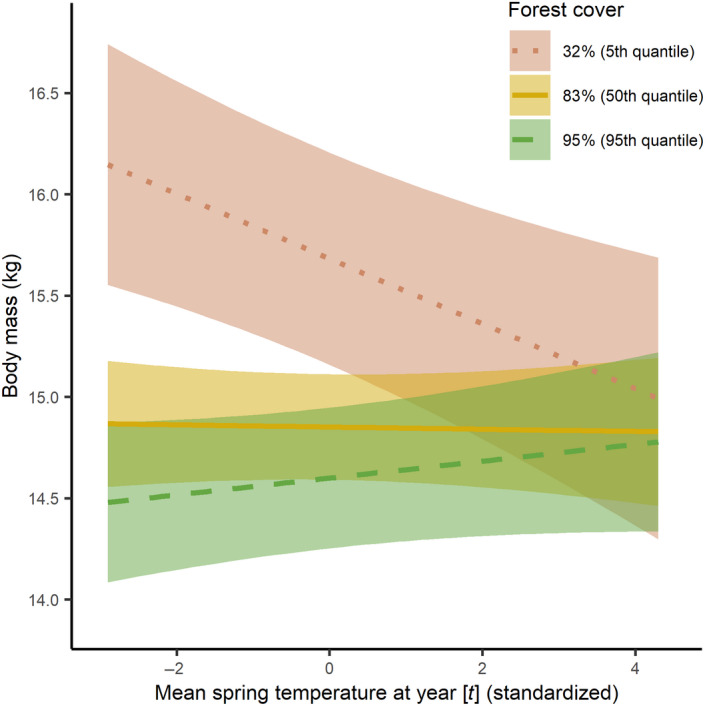
Marginal effects of the interaction between spring temperature at year [*t*] and forest cover, fitted to explain variation in yearling chamois body mass in Austria, between 1993 and 2019. The levels of forest cover correspond to the 5th (32% forest cover; red dotted line), 50th (83% forest cover; yellow solid line), and 95th (95% forest cover; green dashed line) quantiles. Shaded areas indicate 95% confidence intervals (all other terms of the model are kept at their mean values). The *x*‐axis indicates the annual deviation (in °C) from the mean spring temperature for the entire study period

The most parsimonious model to explain body mass variation over time at different proportions of forest cover included the predictor variables: sex, quadratic Julian day, density at [*t*], forest cover, snow depth during winter at [*t*], spring temperature at [*t* − 1], summer temperature at [*t* − 1], spring temperature at [*t*], and summer temperature [*t*], as well as the interactions density at [*t*] × snow depth during winter at [*t*], spring temperature at [*t*] × forest cover and spring temperature at [*t* − 1] × forest cover (conditional *R* ^2^ = 0.097, marginal *R* ^2^ = 0.042). However, five other models had ΔAIC ≤ 2 (Table 3) and were thus retained in the candidate set for model averaging. The averaged model included significant positive effects of quadratic Julian day and snow depth during winter at [*t*], significant negative effects of density at [*t*], forest cover, summer temperature at [*t*], and summer temperature at [*t* − 1] on yearling chamois body mass (Table 4). In addition, the averaged model included the significant positive interactions spring temperature at [*t*] × forest cover (Figure 4), spring temperature at [*t* − 1] × forest cover, and the significant positive interaction between density at [*t*] × snow depth during winter at [*t*] (Table 4; Figure S3). Notably, the results (both in terms of models selected and parameter estimates) did not change when using a different geographic (*n* = 49 chamois management units) or temporal (time period 1998–2019) subdivision (Table S3).

**TABLE 3:** Set of the most parsimonious models (ΔAIC ≤ 2) to explain temporal variation in yearling chamois body mass in the Austrian Alps, between 1993 and 2019. The table reports the explanatory variables in the linear predictor, along with their beta estimates, the degrees of freedom (*df*), ΔAIC (difference between model‐specific AIC and lowest AIC), and model weight (Wt). See Table 1 for a description of explanatory variables

Sex	Julianday^2^	Density	%forest	SnowD_wi	TSpr_*t* − 1	Tsum_*t* − 1	TSpr_*t*	Tsum_*t*	TSpr_*t* − 1 × %forest	TSpr_*t* × %forest	TSum_*t* × %forest	TSum_*t* − 1 × %forest	Density × SnowD_wi	SnowD_wi × %forest	*df*	ΔAIC	Weight
0.59	1.24	−0.12	−0.30	0.21	0.06	−0.09	0.006	−0.13	0.04	0.06			0.16		18	0.0	0.30
0.59	1.24	−0.13	−0.29	0.21	0.05	−0.09	−0.007	−0.13	0.04	0.06		0.02	0.15		19	1.1	0.17
0.59	1.24	−0.13	−0.29	0.22	0.05	−0.09	−0.008	−0.13	0.03	0.05	0.01	0.03	0.15		17	1.2	0.16
0.59	1.24	−0.12	−0.30	0.21	0.06	−0.09	0.007	−0.13	0.04	0.05	0.01		0.16		19	1.4	0.14
0.59	1.24	−0.13	−0.29	0.22	0.05	−0.09	0.008	−0.13	0.04	0.06	0.02		0.15	−0.02	19	1.8	0.12
0.59	1.24	−0.12	−0.30	0.21	0.06	−0.09	0.006	−0.13	0.04	0.06			0.16	−0.02	19	1.9	0.11

**TABLE 4:** Average parameter estimates of the models with ΔAIC ≤ 2, fitted to investigate climate‐induced body mass variation of yearling chamois harvested in the study area between 1993 and 2019. The table reports variables used in the analysis, averaged beta estimates, standard errors (SE), *t*‐score (*t*), upper and lower 95% confidence interval (CI), and the *p* value

Variable	Estimate	SE	*t*	CI_0.025_	CI_0.975_	*p*
Intercept	13.44	0.15	88.58	13.15	13.74	<0.001
Sex (male)	0.59	0.04	16.15	0.51	0.66	<0.001
Julianday^2^	1.24	0.12	10.01	1.00	1.48	<0.001
Density	−0.12	0.02	5.95	−0.16	−0.08	<0.001
%forest	−0.30	0.10	3.02	−0.49	−0.10	0.003
SnowD_wi	0.21	0.09	2.26	0.03	0.39	0.024
TSum_*t*	−0.13	0.04	3.69	−0.20	−0.06	<0.001
TSum_*t* − 1	−0.09	0.04	−2.48	−0.16	−0.02	0.013
TSpr_*t* − 1 × %forest	0.04	0.03	2.70	0.01	0.07	0.007
TSpr_*t* × %forest	0.06	0.02	3.67	0.03	0.09	<0.001
Density × SnowD_wi	0.16	0.07	2.12	0.01	0.30	0.034

## 4 DISCUSSION

Our results confirmed prediction (a) that is, a temporal decline of yearling chamois body mass during the study period. This decline, however, was less pronounced in areas with greater proportion of forest cover, and for chamois living in forested areas only, no significant temporal change in body mass was expected. The interaction between spring temperature at [*t*] and [*t* − 1] and forest cover contributed significantly to explain habitat‐dependent variance in body mass, thereby supporting prediction (b), a less pronounced effect of increasing spring–summer temperatures on body mass variation with increasing forest cover.

The decreasing temporal trend in chamois body mass is in line with the results of previous studies on several taxa (Gardner et al., 2011). For example, a decline in body mass over time related to climate change was found in four different passerine species (Yom‐Tov, 2001), in North American birds (Van Buskirk et al., 2010), in Soay sheep *Ovis aries* (Ozgul et al., 2009), and Canadian lynx (Yom‐Tov et al., 2007). Climatic effects are expected to be particularly evident in mountainous and temperate habitats (Diaz & Bradley, 1997; Turco et al., 2015), and temperature‐induced changes in body conditions have been reported in several ungulates inhabiting temperate environments at higher elevations, for example, in bighorn sheep *Ovis canadensis*, Alpine ibex *Capra ibex*, mountain goats *Oreamnus americanus* (Pettorelli et al., 2007) or at higher latitudes, for example, Moose *Alces alces*, alpine reindeer *Rangifer tarandus* (Borowik et al., 2020; Herfindal et al., 2006; Pettorelli et al., 2005). Previous findings consistently reported a negative temporal trend in body mass related to increasing temperatures also in chamois, possibly due to low food quality and quantity with increased temperature (Mason, Apollonio, et al., 2014; Rughetti & Festa‐Bianchet, 2012): our results, however, provide evidence for differential temporal trends as a function of habitat type.

To date, most studies on climate‐related mountain ungulate biology focused on populations living in areas above the tree line (e.g., Brivio et al., 2019; Chirichella et al., 2021; Lovari et al., 2020; Rattenbury et al., 2018). By focusing on a variety of areas at different elevations, our study allowed to disentangle body mass variations in relation to different proportion of forest cover within mountain ranges, suggesting a temperature‐buffering effect of forests. Our data did not include individual‐specific coordinates, and our modeling approach allowed to only partly account for spatial correlation; parameter estimates with *p* values close to 0.05 (e.g., the interaction between density and snow cover) therefore need to be treated with caution, as unmodeled residual correlation might increase the chance of a Type I error. The negative relationship between spring temperatures in the current year and body mass was mitigated by increasing forest cover and disappeared in entirely forested mountain ranges. Additionally, we found a similar result for spring temperature in the year of birth, suggesting that the negative relationship between spring temperature in the year of birth and body mass is also buffered by increasing forest cover. These results support the role of forests as a potential shelter against the effects of increasing temperatures on key life‐history traits in ungulates (Melin et al., 2014). The biological explanation for habitat‐specific differences in body mass variation is unclear, though it might be related to foraging opportunities and costs for thermoregulation (Bubenik, 1984). The low variance explained by the model might reflect this uncertainty. Increasing temperatures in Alpine areas have a negative effect on chamois foraging activity (Brivio et al., 2016), leading to reduced diurnal activity and increased nocturnal activity, when temperatures are generally lower (Grignolio et al., 2018). As opposed to populations living in open areas, a more even distribution of activity throughout the day during warm periods has been suggested for forest‐dwelling chamois, possibly owing to the thermal buffering and cooler temperatures offered by canopy cover (Breidermann, 1967; Šprem et al., 2015). This, in turn, might allow chamois to maintain a certain stability in time spent foraging within forests despite increasing temperatures. While forage quality (i.e., more nutritious and palatable plants) in general is expected to be better at higher elevations (i.e., in Alpine meadows) in summer (Van Soest, 1994), the climate‐dependent variation in quality and availability of forage is expected to be less pronounced in forested areas (Norris et al., 2012). Furthermore, climate change is substantially altering within‐forest dynamics, for example, by increasing natural disturbances, both in frequency and size (Senf & Seidl, 2021). Natural disturbances in forested areas may reduce the tree cover, but at the same time also result in improved forage opportunities within forested areas (Vavra & Riggs, 2010). Finally, the energetic costs for thermoregulation may also be mitigated in forests, since forests are known to significantly affect air temperature: in montane regions, for example, variation in maximum temperature is explained more by canopy cover than by elevation (Frey et al., 2016).

The significant negative relationship of density and body mass broadly supports the results of other studies, which is mainly explained by competition for limited resources (Festa‐Bianchet et al., 2019; Toïgo et al., 2006). We also found a positive correlation between snow depth and body mass as well for the interaction between density and snow cover. This may be explained by the fact that smaller individuals experience higher mortality in unfavorable environmental conditions, such as high snow depth, and especially at high densities, which, in turn, leads to higher average body mass of the surviving individuals (Lindstedt & Boyce, 1985).

Long‐term declines in body mass may represent an adaptation to climate change (Gardner et al., 2011), though it has been suggested that climate‐induced body mass decline may negatively affect population dynamics (Boutin & Lane, 2014; Chirichella et al., 2021). Over the long term, however, climate change may impose further threats, such as a decrease in suitable habitats at higher elevations, which, in turn, may cause a numerical decline of chamois populations (e.g., in Apennine chamois *Rupicapra pyrenaica* *ornata*, Lovari et al., 2020). Lower spring temperatures delay the snowmelt at high elevations which, in turn, delays the onset of plant growth and prolongs access to high‐quality vegetation (Aublet et al., 2009). Although climate change leads to a rise in the tree line, this effect may not keep pace with habitat loss induced by climate change in the Alpine zone (Schwager & Berg, 2019). Nonetheless, our results suggest that chamois might be able to take advantage of forested areas at lower elevations (Schröder & von den Marlsburg, 1982; Zeiler, 2012) to mitigate the effects of increasing temperatures, provided that these habitats are suitable for the species (i.e., with high presence of rocky areas, von Elsner‐Schack, 1985). Increasing use of forest habitats, for example, has been shown in chamois populations in the Tatra mountains (Ciach & Pęksa, 2019). Forest colonization by chamois will also depend on the presence and distribution of large carnivores, on the occurrence of suitable food resources and/or resulting resource competition with other ungulates (Corlatti et al., 2019) as well as on the occurrence of conflicts with human activities, such as forestry or recreation (cf. Ciach & Pęksa, 2019; Schnidrig‐Petrig & Ingold, 2001).

Overall, the future of chamois will not only depend on how the species will cope with changes in climatic conditions, but with a combination of different factors, including among other things, climate change (Lovari et al., 2020), land use (Ciach & Pęksa, 2019), hunting (Skonhoft et al., 2002), interspecific competition (Corlatti et al., 2019; Ferretti et al., 2015), and disease outbreaks (Rossi et al., 2019). The assessment of chamois population dynamics scenarios should thus consider the plasticity of the species with respect to all the aforementioned factors. Investigating the bio‐ecology of the species in forested areas would allow to parametrize life‐history traits of chamois populations more efficiently across different habitats, both above and below the tree line, and at different environmental conditions. The ability to cope with different ecological factors is expected to affect several life‐history parameters such as fecundity, survival (Bleu et al., 2015), density, dispersal (Bowler & Benton, 2005), and ultimately the mating system (Clutton‐Brock & Harvey, 1978). This study strongly suggests a buffering effect of forests against climate change impact, an aspect to be considered when modeling future demography and distribution ranges of plastic species under pressure of rising temperatures.

## AUTHORS’ CONTRIBUTIONS

R.R., L.C., A.Z., and H.Z. conceived the idea for the manuscript. R.R. and H.Z. acquired the data. R.R., L.C., and A.Z. defined the final analysis; R.R. and L.C. analyzed the data; R.R. wrote the first manuscript draft; R.R., A.Z., H.Z., K.H., and L.C. commented, discussed, and finalized the manuscript. Drawings by H.Z.

## Supporting information

Appendix S1

## Data Availability

The data that support the findings of this study are available from the corresponding author upon reasonable request.
