# Supplementary material for: Forests buffer the climate‐induced decline of body mass in a mountain herbivore
Source: Glob Chang Biol. 2021 Jun 3;27(16):3741–52. doi: 10.1111/gcb.15711 (PMC8361913; doi:10.1111/gcb.15711)

**Supplementary Material**

**Forests buffer the climate-induced decline of body mass in a mountain herbivore**

Reiner, R., Zedrosser, A., Zeiler, H., Hackländer, K., Corlatti, L.

**Appendix 1**

**Table S1:** Mountain ranges, habitat metrics, yearling chamois harvest data and climatic data and in the provinces of Salzburg, Styria and Carinthia, Austria, 1993-2019

| Mountain range ID | Mountain range | Total suitable habitat | Forest cover | Number of chamois | | Mean body mass (eviscerated, without head) | | Mean spring temperature | Mean summer temperature | Mean Snow depth winter |
| --- | --- | --- | --- | --- | --- | --- | --- | --- | --- | --- |
|  |  | (ha) | (%) | Males, *n* | Females, n | Males, kg | Females, kg | (°C) | (°C) | cm |
| 9 | Lofer and Leogang Mountains | 12,817 | 77 | 140 | 119 | 13.9 | 13.7 | 11.1 | 18.1 | 102 |
| 10 | Berchtesgaden Alps | 52,162 | 65 | 589 | 575 | 14.3 | 13.8 | 12.0 | 18.8 | 91 |
| 11 | Chiemgau Alps | 10,262 | 85 | 78 | 85 | 14.9 | 13.7 | 14.1 | 20.7 | 82 |
| 12 | Salzburger Schieferalpen | 37,478 | 87 | 132 | 144 | 15.1 | 14.8 | 13.2 | 19.7 | 50 |
| 13 | Tennen Mountain | 29,714 | 70 | 203 | 232 | 14.1 | 13.3 | 12.0 | 18.8 | 82 |
| 14 | Dachstein Mountains | 44,340 | 80 | 218 | 328 | 14.8 | 14.7 | 10.5 | 17.4 | 83 |
| 15 | Totes Gebirge | 50,087 | 77 | 218 | 243 | 15.3 | 14.8 | 10.3 | 17.1 | 95 |
| 16 | Ennstal Alps | 108,703 | 85 | 371 | 391 | 14.2 | 13.6 | 14.0 | 20.6 | 44 |
| 17a | Salzkammergut Mountains | 69,401 | 92 | 658 | 765 | 15.2 | 14.5 | 13.9 | 20.7 | 70 |
| 18 | Hochschwab | 93,708 | 85 | 690 | 803 | 14.9 | 14.4 | 14.0 | 20.7 | 46 |
| 19 | Mürzsteg Alps | 38,285 | 93 | 456 | 479 | 13.9 | 13.8 | 13.5 | 20.4 | 23 |
| 20 | Rax and Schneeberg | 6,130 | 91 | 103 | 143 | 14.0 | 13.5 | 13.5 | 20.4 | 20 |
| 21 | Ybbstal Alps | 31,488 | 96 | 306 | 302 | 15.0 | 14.5 | 13.0 | 19.8 | 64 |
| 34 | Kitzbühel Alps | 37,975 | 60 | 274 | 328 | 16.3 | 15.8 | 11.4 | 18.2 | 75 |
| 35 | Zillertal Alps | 11,444 | 28 | 27 | 23 | 16.1 | 14.9 | 6.2 | 13.5 | 130 |
| 36 | Venediger Group | 26,722 | 24 | 77 | 84 | 14.9 | 14.0 | 6.4 | 13.6 | 111 |
| 39 | Granatspitz Group | 16,760 | 34 | 74 | 48 | 16.0 | 14.9 | 8.4 | 15.6 | 89 |
| 40 | Glockner Group | 40,318 | 27 | 305 | 305 | 16.5 | 15.7 | 8.4 | 15.5 | 88 |
| 42 | Goldberg Group | 35,698 | 40 | 191 | 209 | 15.6 | 14.7 | 8.5 | 15.5 | 90 |
| 44 | Ankogel Group | 43,271 | 47 | 223 | 314 | 14.8 | 14.3 | 8.9 | 15.8 | 76 |
| 45a | Radstadt Tauern | 63,185 | 61 | 468 | 476 | 15.0 | 14.3 | 9.4 | 16.6 | 46 |
| 45b | Schladming Tauern | 89,686 | 63 | 478 | 514 | 16.2 | 15.4 | 9.7 | 16.9 | 53 |
| 45c | Rottenmann and Wölz Tauern | 95,460 | 73 | 452 | 454 | 15.2 | 14.4 | 10.2 | 17.2 | 52 |
| 45d | Seckau Tauern | 45,736 | 83 | 239 | 316 | 15.9 | 15.3 | 10.7 | 17.7 | 48 |
| 46a | Gurktal Alps | 39,082 | 89 | 189 | 295 | 15.2 | 15.1 | 10.7 | 17.9 | 29 |
| 46b | Lavanttal Alps | 154,322 | 95 | 1,716 | 2,268 | 15.0 | 15.0 | 13.7 | 20.8 | 17 |
| 47 | Prealps East of the Mur | 75,404 | 99 | 398 | 479 | 14.3 | 13.8 | 13.9 | 21.0 | 14 |
| 56 | Gailtal Alps | 12,456 | 95 | 282 | 296 | 13.6 | 13.1 | 14.0 | 21.4 | 30 |

**Table S2:** Total and annual number of harvested yearling chamois in mountain ranges of the provinces Salzburg, Styria and Carinthia, Austria, 1993-2019. Shaded cells indicate years where no data have been reported for the corresponding mountain range.

| Mountain range ID | Total | 1993 | 1994 | 1995 | 1996 | 1997 | 1998 | 1999 | 2000 | 2001 | 2002 | 2003 | 2004 | 2005 | 2006 | 2007 | 2008 | 2009 | 2010 | 2011 | 2012 | 2013 | 2014 | 2015 | 2016 | 2017 | 2018 | 2019 |
| --- | --- | --- | --- | --- | --- | --- | --- | --- | --- | --- | --- | --- | --- | --- | --- | --- | --- | --- | --- | --- | --- | --- | --- | --- | --- | --- | --- | --- |
| 9 | 259 |  |  |  |  |  | 18 | 12 | 7 | 17 | 9 | 16 | 17 | 6 | 7 | 6 | 11 | 6 | 12 | 9 | 11 | 12 | 11 | 16 | 16 | 20 | 10 | 10 |
| 10 | 1,164 |  |  |  |  |  | 58 | 56 | 50 | 64 | 61 | 66 | 71 | 57 | 46 | 60 | 53 | 41 | 55 | 61 | 42 | 31 | 38 | 55 | 62 | 56 | 42 | 39 |
| 11 | 163 |  |  |  |  |  | 10 | 3 | 10 | 3 | 8 | 9 | 9 | 8 | 2 | 5 | 9 | 9 | 7 | 8 | 5 | 8 | 7 | 13 | 9 | 11 | 8 | 2 |
| 12 | 276 | 1 | 1 | 2 | 1 | 2 | 14 | 7 | 5 | 9 | 11 | 11 | 6 | 12 | 14 | 12 | 11 | 12 | 14 | 18 | 15 | 12 | 20 | 13 | 20 | 14 | 11 | 8 |
| 13 | 435 |  |  |  |  |  | 22 | 17 | 26 | 32 | 35 | 28 | 42 | 17 | 24 | 17 | 15 | 11 | 16 | 10 | 13 | 14 | 18 | 14 | 20 | 18 | 13 | 13 |
| 14 | 546 | 17 | 26 | 16 | 21 | 18 | 24 | 21 | 18 | 27 | 33 | 37 | 23 | 17 | 18 | 38 | 25 | 13 | 28 | 14 | 11 | 16 | 14 | 11 | 20 | 13 | 11 | 16 |
| 15 | 461 | 22 | 29 | 14 | 16 | 36 | 38 | 17 | 23 | 27 | 19 | 23 | 23 | 11 | 12 | 20 | 13 | 13 | 19 | 16 | 8 | 7 | 8 | 11 | 8 | 13 | 9 | 6 |
| 16 | 762 | 47 | 51 | 49 | 53 | 58 | 53 | 29 | 23 | 40 | 38 | 37 | 27 | 25 | 11 | 20 | 13 | 11 | 12 | 21 | 14 | 21 | 22 | 9 | 22 | 22 | 16 | 18 |
| 17a | 1,423 |  |  |  |  |  | 73 | 40 | 67 | 71 | 75 | 71 | 65 | 75 | 45 | 57 | 74 | 55 | 59 | 52 | 69 | 64 | 67 | 84 | 90 | 57 | 54 | 59 |
| 18 | 1,493 | 77 | 89 | 75 | 62 | 78 | 82 | 49 | 61 | 74 | 78 | 93 | 84 | 34 | 25 | 52 | 55 | 21 | 41 | 51 | 26 | 38 | 54 | 30 | 47 | 47 | 41 | 29 |
| 19 | 935 | 27 | 49 | 32 | 47 | 46 | 62 | 40 | 37 | 47 | 46 | 39 | 37 | 16 | 26 | 34 | 49 | 20 | 36 | 30 | 23 | 18 | 21 | 23 | 31 | 37 | 32 | 30 |
| 20 | 246 | 6 | 12 | 11 | 16 | 13 | 11 | 9 | 7 | 10 | 18 | 11 | 8 | 4 | 2 | 6 | 8 | 1 | 9 | 8 | 2 | 10 | 9 | 9 | 14 | 15 | 11 | 6 |
| 21 | 608 | 19 | 32 | 29 | 27 | 32 | 38 | 19 | 22 | 47 | 53 | 33 | 24 | 20 | 12 | 17 | 21 | 14 | 12 | 18 | 14 | 16 | 10 | 9 | 15 | 18 | 24 | 13 |
| 34 | 602 |  |  |  |  |  | 34 | 25 | 31 | 25 | 36 | 43 | 27 | 29 | 23 | 25 | 19 | 17 | 15 | 32 | 21 | 21 | 32 | 29 | 41 | 39 | 24 | 14 |
| 35 | 50 |  |  |  |  |  | 8 | 4 | 6 | 8 | 4 | 3 | 2 | 3 | 1 | 1 | 0 | 0 | 2 | 0 | 0 | 0 | 2 | 0 | 0 | 5 | 0 | 1 |
| 36 | 161 |  |  |  |  |  | 17 | 19 | 16 | 18 | 20 | 13 | 12 | 4 | 4 | 2 | 5 | 3 | 1 | 2 | 2 | 3 | 4 | 3 | 1 | 7 | 2 | 3 |
| 39 | 122 |  |  |  |  |  | 10 | 8 | 8 | 7 | 13 | 9 | 6 | 10 | 3 | 2 | 4 | 1 | 4 | 2 | 2 | 2 | 6 | 7 | 5 | 7 | 5 | 1 |
| 40 | 610 |  |  |  |  |  | 46 | 35 | 42 | 36 | 33 | 33 | 32 | 29 | 18 | 16 | 12 | 20 | 19 | 25 | 19 | 21 | 37 | 35 | 26 | 25 | 30 | 21 |
| 42 | 400 |  |  |  |  |  | 18 | 20 | 25 | 28 | 32 | 21 | 29 | 24 | 17 | 22 | 18 | 17 | 15 | 15 | 13 | 10 | 14 | 14 | 11 | 15 | 12 | 10 |
| 44 | 537 |  |  |  |  |  | 43 | 46 | 49 | 42 | 30 | 40 | 33 | 34 | 24 | 27 | 26 | 17 | 20 | 16 | 13 | 11 | 17 | 7 | 12 | 17 | 6 | 7 |
| 45a | 944 |  |  |  |  |  | 50 | 63 | 51 | 60 | 55 | 53 | 53 | 67 | 44 | 45 | 46 | 35 | 34 | 35 | 31 | 26 | 34 | 35 | 37 | 34 | 36 | 20 |
| 45b | 992 | 15 | 20 | 10 | 13 | 13 | 58 | 56 | 53 | 59 | 62 | 55 | 58 | 52 | 47 | 54 | 31 | 30 | 41 | 33 | 30 | 30 | 25 | 31 | 28 | 37 | 24 | 27 |
| 45c | 906 | 51 | 60 | 39 | 69 | 52 | 54 | 35 | 45 | 38 | 44 | 32 | 27 | 32 | 22 | 36 | 29 | 17 | 32 | 23 | 31 | 21 | 21 | 22 | 20 | 18 | 16 | 20 |
| 45d | 555 | 37 | 33 | 26 | 46 | 34 | 46 | 36 | 28 | 28 | 33 | 35 | 17 | 22 | 14 | 19 | 14 | 4 | 5 | 4 | 8 | 5 | 11 | 4 | 8 | 12 | 17 | 9 |
| 46a | 484 | 5 | 6 | 7 | 11 | 11 | 16 | 19 | 33 | 37 | 43 | 30 | 26 | 22 | 20 | 15 | 13 | 21 | 15 | 25 | 15 | 14 | 14 | 12 | 13 | 18 | 12 | 11 |
| 46b | 3,984 | 110 | 89 | 92 | 109 | 91 | 117 | 126 | 117 | 128 | 125 | 148 | 129 | 136 | 145 | 165 | 163 | 153 | 160 | 145 | 147 | 172 | 170 | 161 | 223 | 219 | 224 | 220 |
| 47 | 877 | 22 | 21 | 31 | 24 | 33 | 29 | 26 | 24 | 24 | 34 | 25 | 27 | 21 | 24 | 28 | 31 | 23 | 37 | 29 | 36 | 40 | 35 | 45 | 47 | 55 | 49 | 57 |
| 56 | 578 | 0 | 1 | 0 | 18 | 18 | 19 | 19 | 16 | 23 | 25 | 27 | 27 | 19 | 26 | 20 | 17 | 34 | 36 | 37 | 34 | 25 | 31 | 28 | 17 | 21 | 20 | 20 |

**Table S3:** Model fitted to explain temporal variation in yearling chamois body mass in the Austrian Alps, between 1998 and 2019. The table reports variables used in the analysis, beta estimates, standard errors (SE), t-score (t), upper and lower 95% confidence interval (CI) and *p*-value.

| **Variable** | ***Estimate*** | ***SE*** | ***t*** | ***CI_0.025_*** | ***CI_0.975_*** | ***p*** |
| --- | --- | --- | --- | --- | --- | --- |
| Intercept | 13.54 | 0.15 | 88.87 | 13.25 | 13.84 | < 0.001 |
| Sex (male) | 0.61 | 0.04 | 15.56 | 0.53 | 0.68 | < 0.001 |
| Julianday² | 1.18 | 0.13 | 8.81 | 0.92 | 1.45 | < 0.001 |
| Density | -0.10 | 0.02 | -4.66 | -0.15 | -0.06 | <0.001 |
| %forest | -0.29 | 0.10 | -2.99 | -0.48 | -0.10 | 0.003 |
| SnowD_wi | 0.19 | 0.09 | 2.02 | 0.01 | 0.37 | 0.043 |
| TSum_t | -0.14 | 0.03 | -4.13 | -0.21 | -0.07 | < 0.001 |
| TSum_t-1 | -0.10 | 0.04 | -2.89 | -0.17 | -0.03 | 0.004 |
| Density × SnowD_wi | 0.13 | 0.07 | 1.80 | -0.01 | 0.28 | 0.072 |
| TSpr_t-1 × %forest | 0.04 | 0.02 | 2.27 | 0.00 | 0.07 | 0.023 |
| TSpr_t × %forest | 0.05 | 0.02 | 3.09 | 0.02 | 0.08 | 0.002 |
|  |  |  |  |  |  |  |

**Figure S1:** Linear model of the relationship between population abundance of chamois based on population reconstruction (Reiner et al., 2020) and annual number of harvested chamois for two mountain ranges included in this study. (a) Seckau Tauern Mountains

(*β* = 4.08, 95% CI: 3.04 to 5.12, *p* < 0.001), Styria (1992-2006) and (b) Tennen Mountains (*β* = 4.64, 95% CI: 3.81 to 5.47, *p* < 0.001), Salzburg (1998-2011), Austria. Shaded areas correspond to 95% confidence intervals.

**
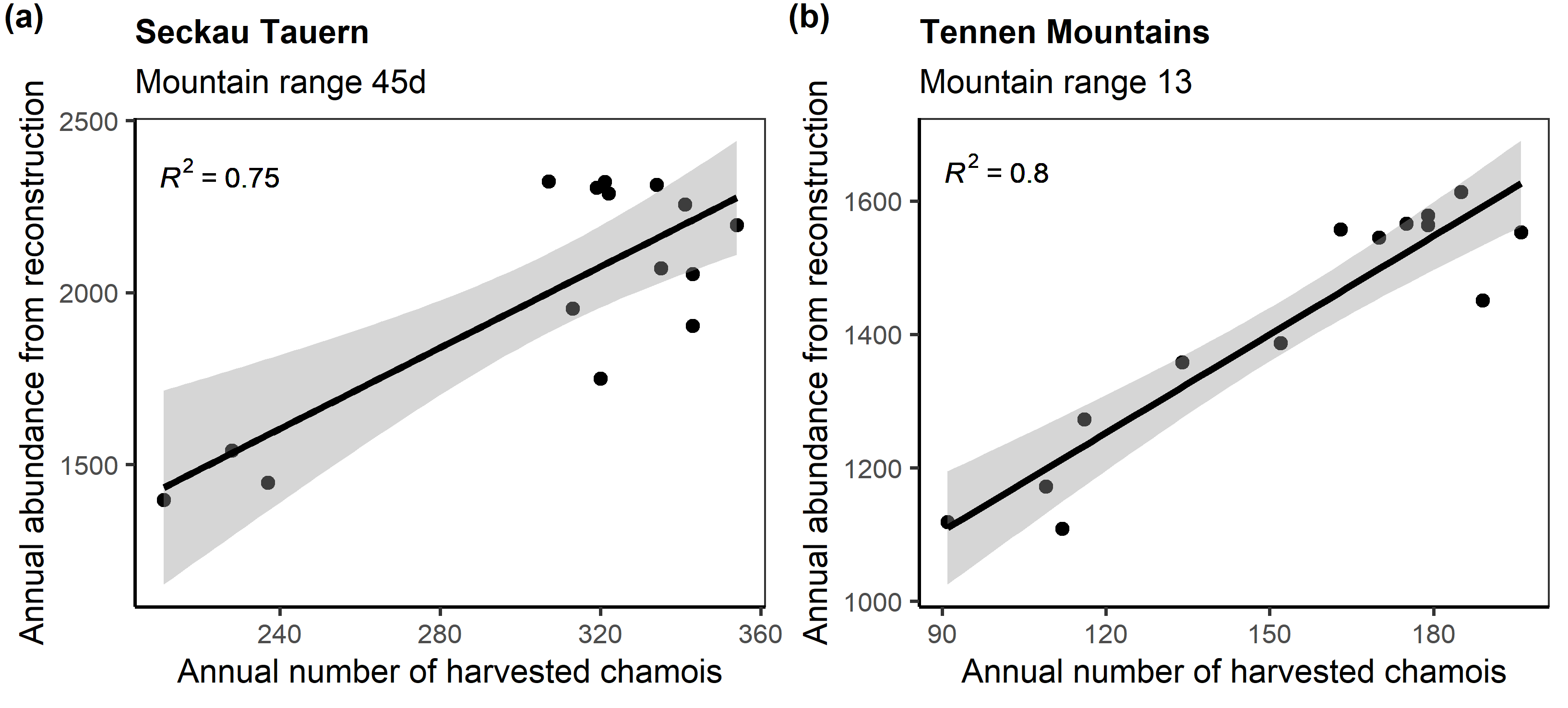
**

**Figure S2:** Relative area of forest cover for 27 mountain ranges in the Eastern Alps, Austria, based on Corine land cover data. The variation over time is mainly caused by slight changes in geometric accuracy of the satellite data (≤ 50 m in 1990, ≤ 25 m in 2000-2012, ≤ 10 m in 2018).


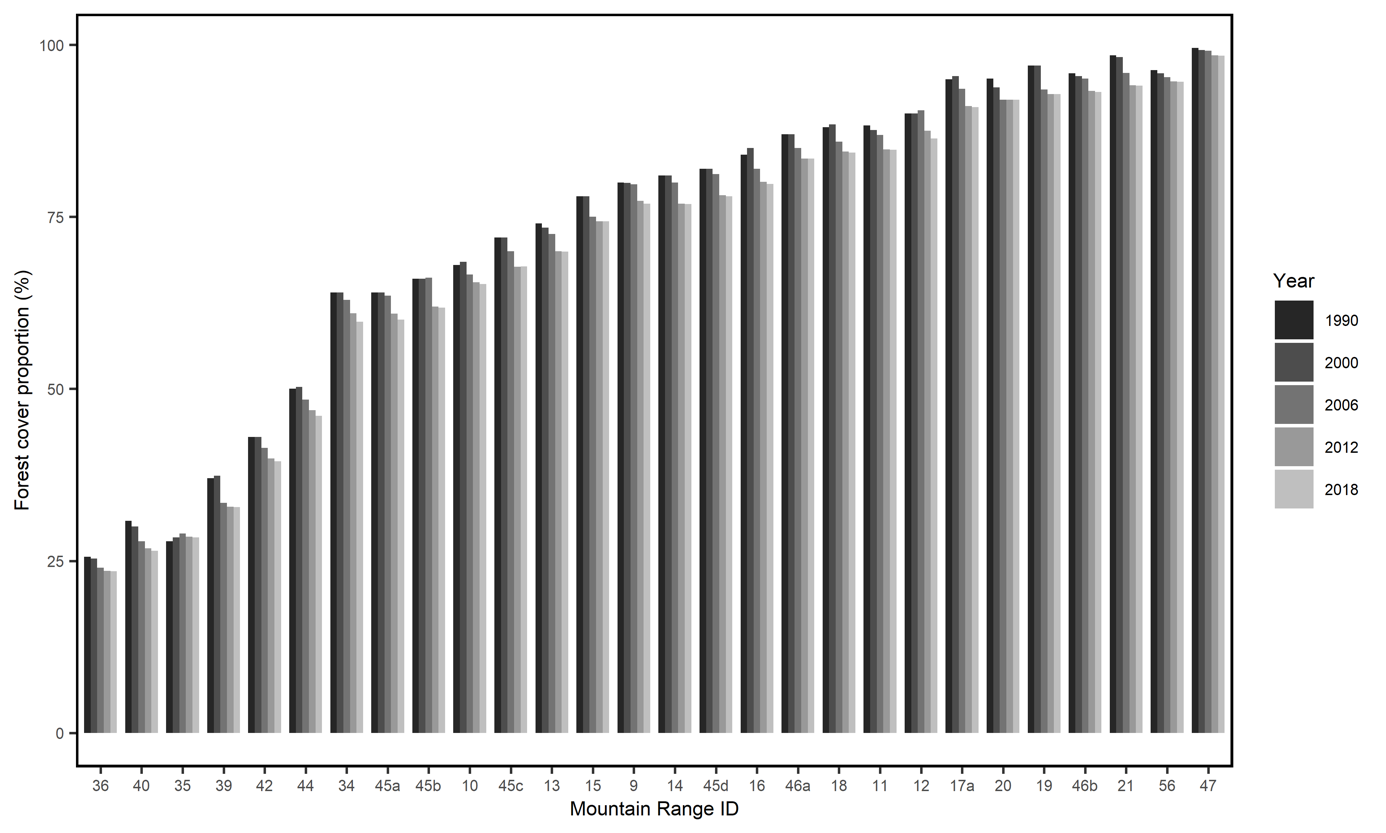


**Figure S3:** Effects of explanatory variables, fitted to explain variation in yearling chamois body mass in Austria, 1993 - 2019: Sex (a), quadratic Julian day (b), mean summer temperature at [t] (c), mean summer temperature at [t-1] (d), interaction between snow depth during winter at [t] and density at [t] (e), and interaction of spring temperature at year [t-1] and forest cover (f). The levels of density at [t] (e) and forest cover (f) correspond to the 5^th^ (dark grey dotted line), 50^th^ (medium grey line) and 95^th^ (light grey dashed line) quantiles. Shaded areas (and bars in (a)) indicate 95% confidential intervals.

**
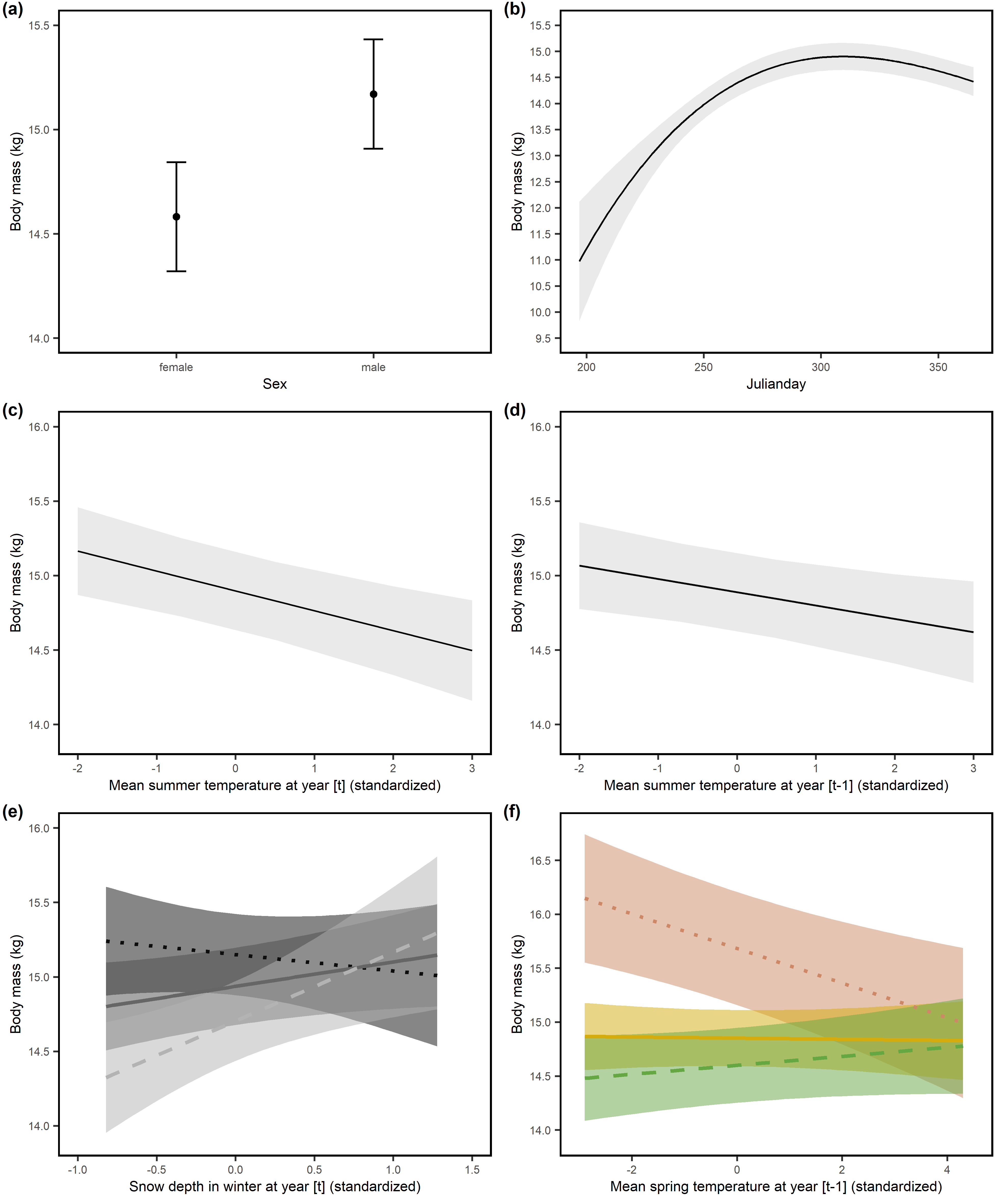
**

**Figure S4:** Residual diagnostics (homogeneity of variance on top, normality on bottom) for LMM fitted to explain temporal (left) and temperature dependent (right) variation in yearling chamois body mass in Austria, 1993 – 2019.


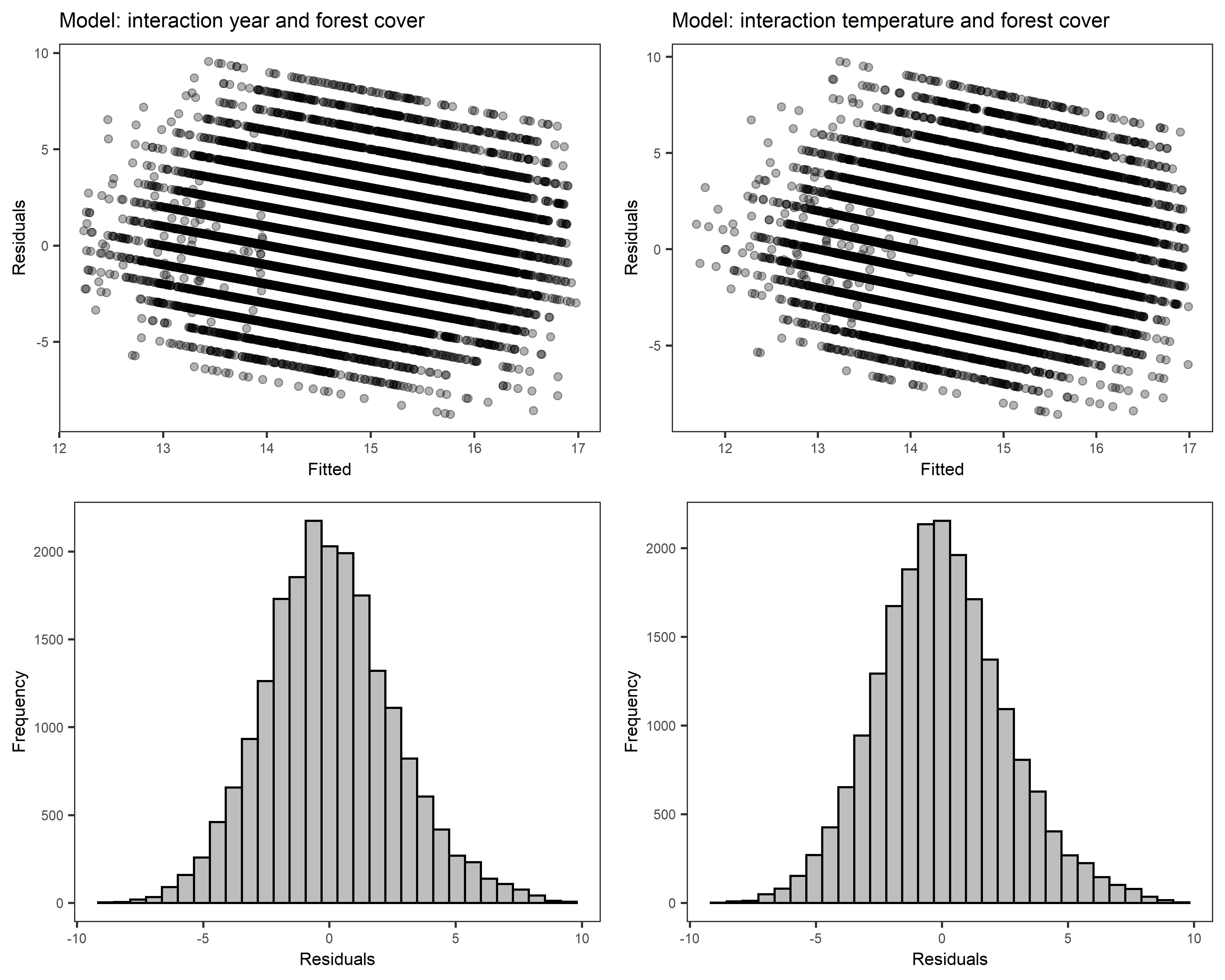

Supplement: Supplementary file 1 — Appendix S1 [file GCB-27-3741-s001.docx]
